# Application of the PDCA Cycle to Improve Quality Control in Pulmonary Function Testing for Early Screening of Chronic Obstructive Pulmonary Disease

**DOI:** 10.1111/crj.70226

**Published:** 2026-08-21

**Authors:** Tingyan Li, Wenyan Hu, Lu Xu, Yongping Gu

**Affiliations:** ^1^ Department of Respiratory Medicine, Linping Campus The Second Affiliated Hospital, Zhejiang University School of Medicine Hangzhou China; ^2^ Department of General Practice, Linping Campus The Second Affiliated Hospital, Zhejiang University School of Medicine Hangzhou China; ^3^ Community Health Service Center of Qiaosi Sub‐district Hangzhou China

**Keywords:** chronic obstructive pulmonary disease, PDCA, pulmonary function testing, quality control

## Abstract

**Objective:**

This study aimed to improve the quality of pulmonary function testing (PFT) used for early screening of chronic obstructive pulmonary disease (COPD) within a regional medical consortium by applying the Plan Do Check Act (PDCA) quality improvement framework.

**Methods:**

This study was conceptualized as a quality improvement (QI) initiative and is documented in compliance with the SQUIRE 2.0 (Standards for Quality Improvement Reporting Excellence) guidelines. A baseline situational assessment within the medical consortium identified insufficient expiratory effort as the primary factor affecting PFT quality. Subsequent root cause analysis indicated that inadequate professional training, absence of continuous supervision, and lack of standardized pre‐examination procedures were the main contributing factors. Based on these findings, targeted quality improvement measures were implemented using the PDCA cycle. These measures included structured training programs, optimization of testing processes, and establishment of a dedicated quality control team. PFT qualification rates were compared before and after implementation. Additionally, a non‐PDCA consortium in the same region that did not apply PDCA‐based initiatives served as a comparison group, and qualification rates between the two consortia were evaluated after 6 months.

**Results:**

In the PDCA consortium, the PFT qualification rate increased from 49.38% prior to implementation to 95.08% following implementation and remained consistently above 95%. In contrast, the non‐PDCA consortium achieved a maximum qualification rate of only 55.78% during the same period.

**Conclusion:**

Application of the PDCA framework effectively optimized PFT procedures and improved overall testing quality within the PDCA consortium.

## Graphical abstract

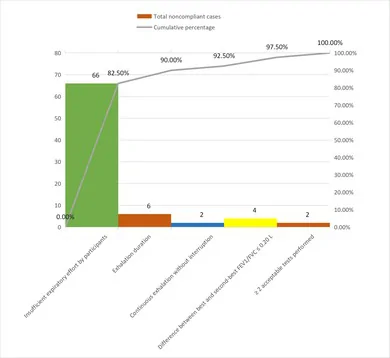

**Abbreviations**
COPD: chronic obstructive pulmonary disease
PDCA: Plan‐Do‐Check‐Act Cycle

## 1 Background

Chronic obstructive pulmonary disease (COPD) is a heterogeneous pulmonary condition characterized by persistent airflow limitation resulting from structural abnormalities of the airways and/or alveoli, and is commonly accompanied by chronic respiratory symptoms [1]. As of 2021, approximately 100 million individuals in China were affected by COPD. Within this context, the Healthy China Action (2019 to 2030) initiative explicitly incorporates pulmonary function screening into routine health examinations for individuals aged 40 years and older. It emphasizes the strengthening of pulmonary function testing (PFT) capabilities of primary healthcare institutions and underscores the central role of PFT in the prevention and management of respiratory diseases [2]. Evidence from international research further supports the necessity of increasing the frequency of PFT among populations at high risk for COPD to facilitate early diagnosis and timely intervention [3]. PFT is regarded as the gold standard for the diagnosis of COPD. Although multiple national policies for COPD prevention and control were issued in China in 2020 to promote early screening and standardized management, public awareness of COPD and the population level coverage of PFT remain low, which substantially limits opportunities for early detection and treatment [4, 5].

The quality of PFT depends on strict adherence to standardized quality control procedures. Survey data indicate that failure to meet quality control standards significantly undermines diagnostic reliability. Therefore, improvement of PFT quality is of critical importance. The present quality improvement (QI) study aimed to evaluate the application of the Plan–Do–Check–Act (PDCA) cycle to improve PFT qualification rates and standardize PFT quality within a medical consortium. This initiative is documented in compliance with the SQUIRE 2.0 guidelines for reporting on healthcare quality improvement.

## 2 Materials and Methods

### 2.1 Formation of the PDCA Cycle Management Team

This research was undertaken as a quality improvement initiative utilizing the PDCA (Plan‐Do‐Check‐Act) cycle framework. The study's design and reporting conform to the SQUIRE 2.0 (Standards for Quality Improvement Reporting Excellence) guidelines [6]. The Yongping Chronic Respiratory Disease Management Studio of the First People's Hospital of Linping District functioned as the central coordinating unit for the PDCA cycle management team. A collaborative framework was established with the Qiaosi, Xingqiao, and Yunhe Community Health Service Centers to form a unified PFT quality control team within the PDCA consortium. The team was led by the studio director and consisted of one chief physician, two associate chief physicians, two attending physicians, one physician, one chief nurse, one associate chief nurse, two charge nurses, one nurse, and the director of the Public Health Department. The team composition ensured comprehensive clinical and administrative expertise and a robust organizational framework. Through coordinated implementation across the affiliated community health service centers, the team sought to achieve rapid and effective improvement in PFT quality.

### 2.2 Baseline Assessment

#### 2.2.1 Pre‐Intervention Data Collection

Between April 25, 2022, and June 25, 2022, a total of 81 pulmonary function tests were conducted across the three branch hospitals of the PDCA consortium as part of early COPD screening efforts. The quality grading was automatically generated by the spirometry software in accordance with the 2019 ATS/ERS spirometry standardization guidelines, which categorize test quality from grade A (highest) to grade F based on the number of acceptable maneuvers and the repeatability of FEV_1_ and FVC measurements [7]. This grading framework has been incorporated into the revised Chinese spirometry standardization [8]. Any borderline results were reviewed and validated by an experienced respiratory physician.

**FIGURE 1:**
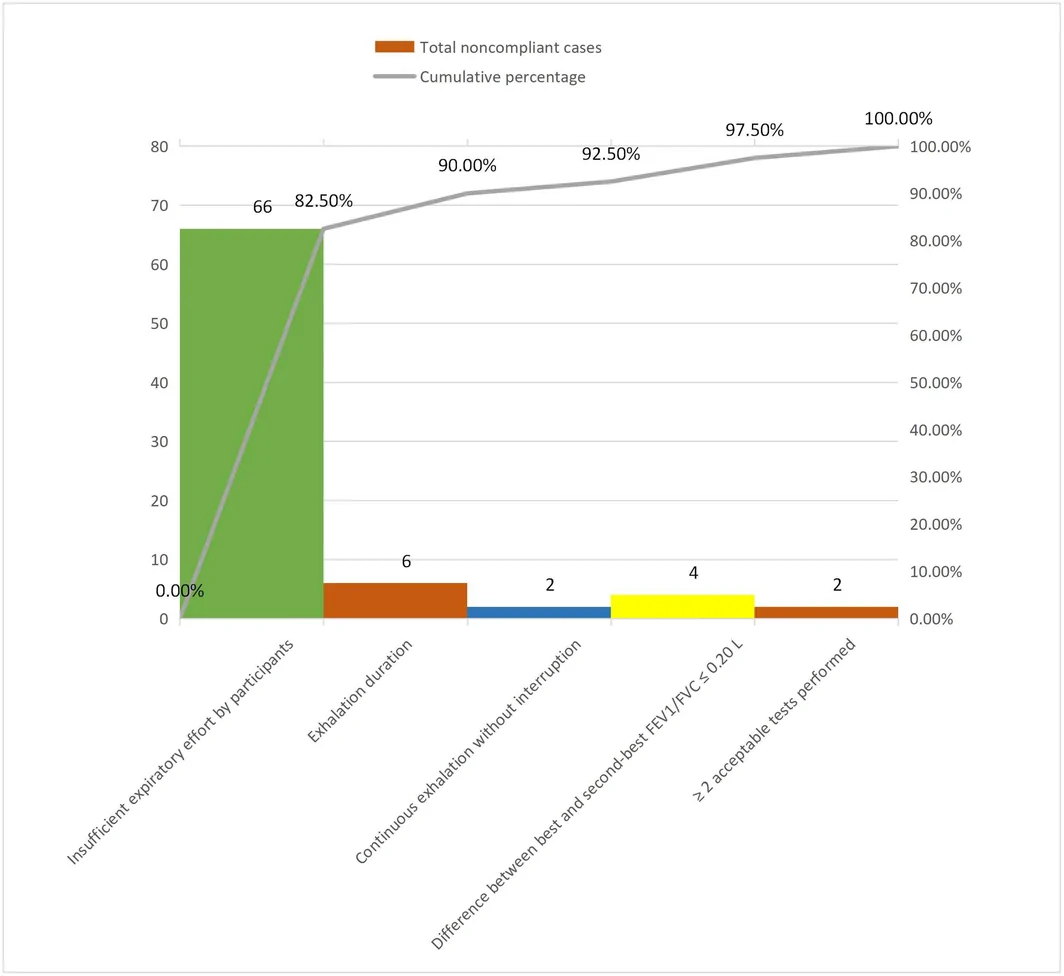
Pareto analysis of noncompliant items in pulmonary function tests before PDCA cycle implementation.

The Results section provides a detailed account of the qualification rates and frequency distribution of noncompliant items for both consortia during the pre‐intervention period (refer to Table 1 and Table 2). To further delineate priority targets for quality enhancement, a Pareto analysis was conducted, focusing on the frequency of noncompliant items. The outcomes of this analysis, particularly the identification of the primary noncompliant item, are illustrated in the Results section (see Figure 1).

**TABLE 1:** Qualification rates of pulmonary function tests for early chronic obstructive pulmonary disease screening in three branch hospitals of the PDCA‐implementing consortium and hospital X.

Month	Qualified cases		Total cases		Qualification rate (%)	
/	PDCA‐implementing consortium (3 branches)	Hospital X	PDCA‐implementing consortium (3 branches)	Hospital X	PDCA‐implementing consortium (3 branches)	Hospital X
April	10	8	25	20	40.00%	40.00%
May	15	11	27	24	55.56%	45.83%
June	15	12	29	26	51.27%	46.16%
Total	40	31	81	70	49.38%	44.29%

**TABLE 2:** Frequency distribution of noncompliant items in pulmonary function tests for early chronic obstructive pulmonary disease screening.

Items	PDCA‐implementing consortium (3 branches)		Hospital X	
	Total noncompliant cases	Noncompliance rate (%)	Total noncompliant cases	Noncompliance rate (%)
Insufficient expiratory effort by participants	66	82.50%	48	72.72%
Exhalation duration	6	7.50%	8	12.12%
Continuous exhalation without interruption	2	2.50%	3	4.54%
Difference between best and second‐best FEV1/FVC ≤ 0.20 L	4	5.00%	5	7.57%
≥ 2 acceptable tests performed	2	2.50%	2	3.03%

*Note:* Insufficient expiratory effort is defined as an extrapolated volume > 0.15 L or extrapolated volume ratio > 5%.

#### 2.2.2 Pre‐Intervention Workflow Analysis

**FIGURE 2:**
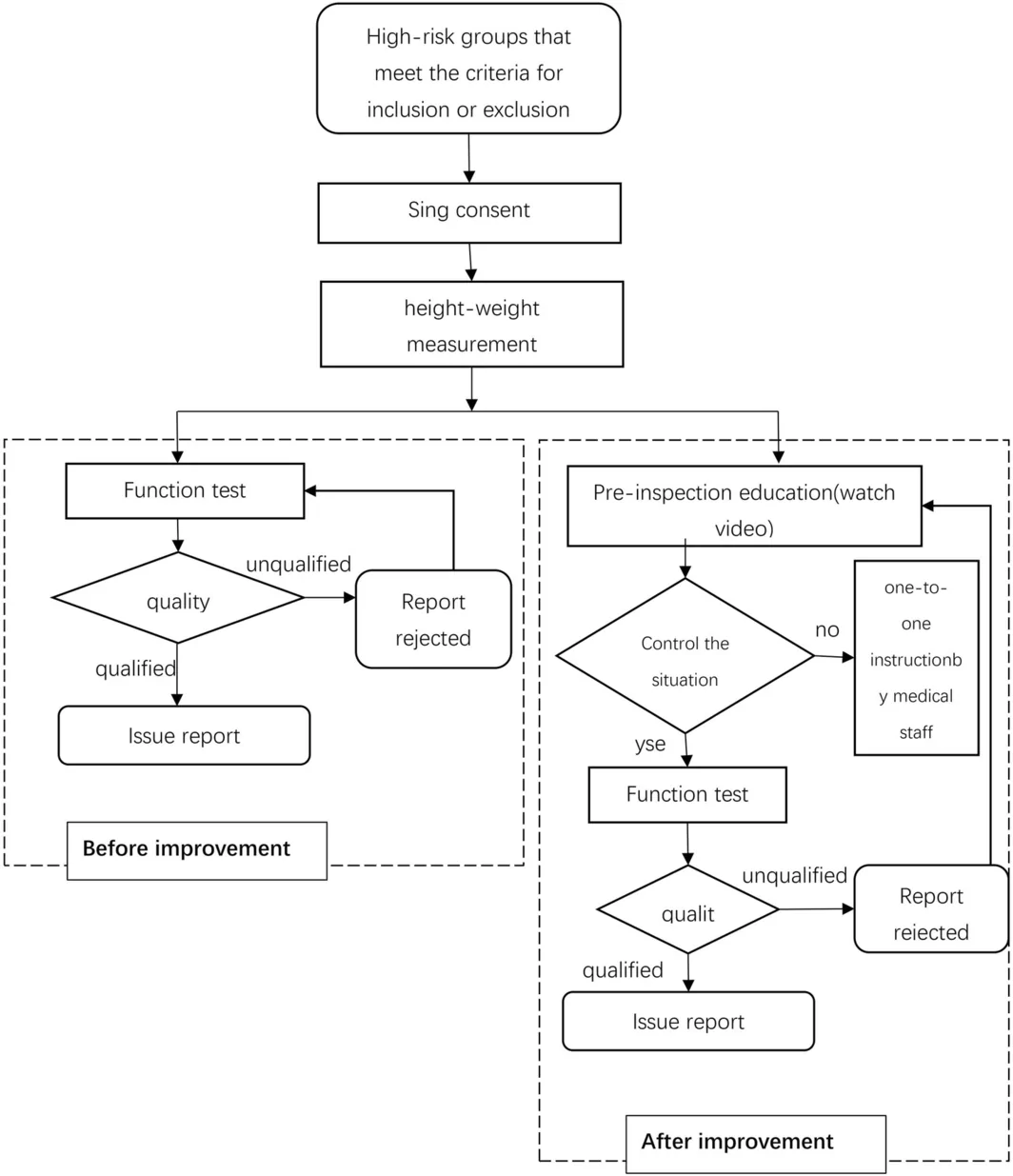
Pulmonary function testing workflow before PDCA cycle implementation.

Individuals identified as high‐risk for COPD included in this study consisted of those with a COPD Screening Questionnaire (COPD SQ) score of 16 or higher or a COPD Population Screener (COPD PS) score of 5 or higher. Exclusion criteria included individuals with severe organ dysfunction, a history of thoracic, abdominal, or ophthalmic surgery within the previous 3 months, and psychiatric or cognitive dysfunction. The detailed workflow for PFT prior to implementation of quality improvement interventions is presented in Figure 2.

#### 2.2.3 Goal Setting

In accordance with the Notice on the Technical Program for Chronic Obstructive Pulmonary Disease Screening in Zhejiang Province issued by the Zhejiang Provincial Health Commission (Zhe Wei Ban Ji Kong [2022] No. 2), the target qualification rate for pulmonary function tests rated at grade C or higher is set at 95%. Based on this standard, the quality improvement target was set at 95%.

### 2.3 Cause Analysis

**FIGURE 3:**
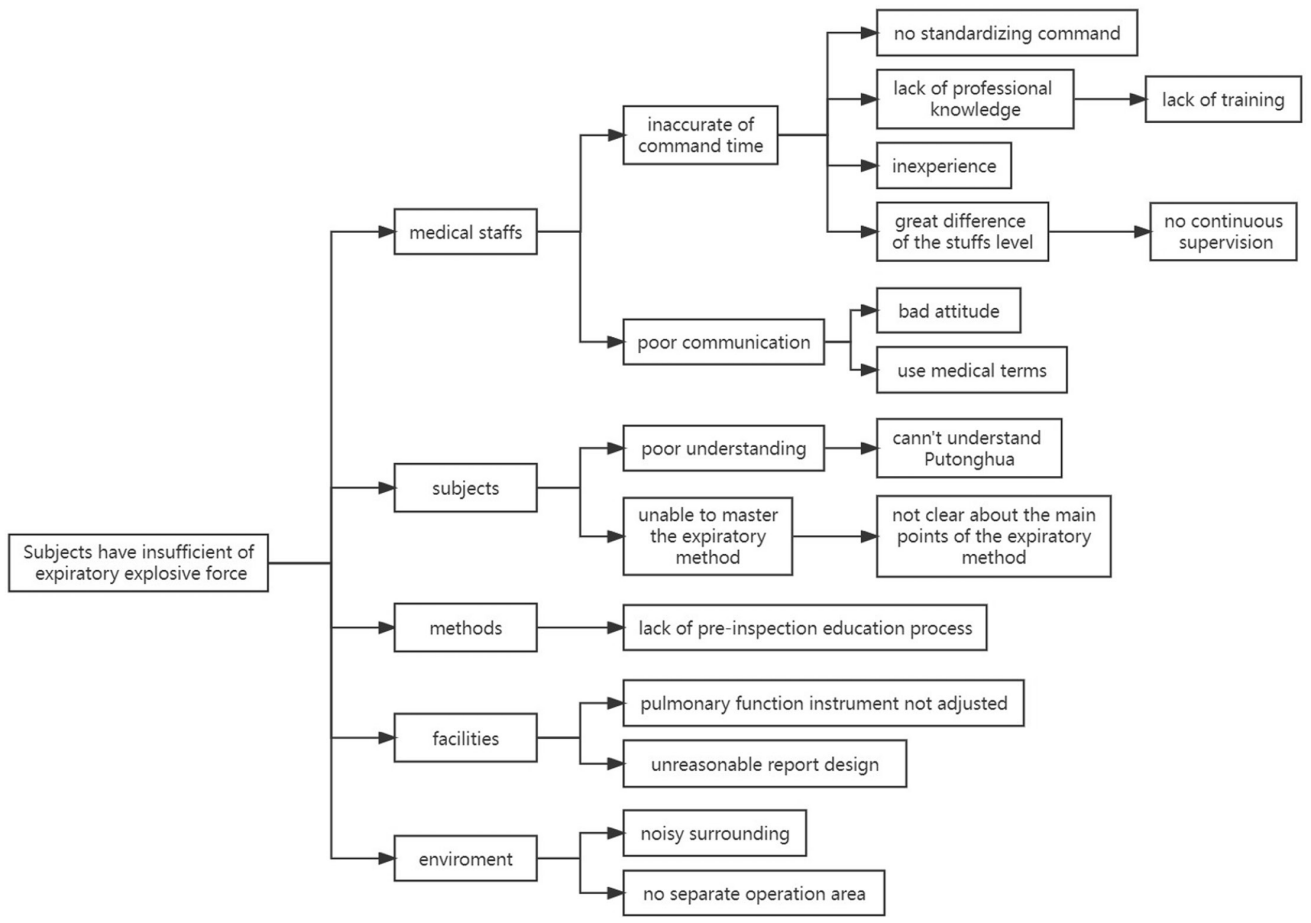
Cause analysis diagram of factors influencing pulmonary function testing quality.

Following analysis of the pre‐intervention data and structured discussions among the PDCA cycle management team, multiple potential factors contributing to substandard PFT quality were identified. The detailed results of this analysis are presented in Figure 3.

### 2.4 Selection of Key Factors and Verification of Root Causes

As delineated in Table 3, team members (designated as A through J) conducted independent evaluations of the identified factors utilizing a five‐point Likert scale, where scores ranged from 1, indicating minimal perceived influence, to 5, indicating maximal perceived influence. This methodology allowed for a maximum potential aggregate score of 50 points. To mitigate potential bias, each evaluator completed their assessments independently before any group discussions occurred. Discrepancies in evaluations were addressed and resolved during a structured consensus meeting, which was facilitated by the team leader. In accordance with the 80/20 principle, key influencing factors were selected, as presented in Table 3. Among the subcomponents associated with insufficient expiratory effort, those with total scores exceeding 40 points were lack of training, absence of continuous supervision, and absence of standardized pre‐examination procedures. These three factors were subsequently verified as root causes using the Three Actuals principle (genchi, genbutsu, genjitsu) through on‐site observation, interviews, and review of existing procedural documentation. The results of this verification process, which identify the three principal root causes of substandard test quality, are presented in the Results section (Table 4).

**TABLE 3:** Scoring results of key factors contributing to insufficient expiratory effort in noncompliant pulmonary function tests.

No.	Cause	A	B	C	D	E	F	G	H	I	J	Total
1	Lack of standardized verbal instructions	4	4	4	4	4	3	3	3	5	4	38
2	Lack of training	3	5	5	3	4	3	4	5	4	5	41
3	Lack of experience	4	4	4	4	4	5	4	3	4	3	39
4	Absence of continuous supervision	4	4	4	5	3	4	4	4	4	5	41
5	Poor attitude	4	3	4	5	3	3	4	4	4	5	39
6	Use of medical terminology	5	4	4	4	3	3	3	3	3	3	35
7	Participants unable to understand Chinese (Mandarin)	2	3	3	5	3	3	3	4	3	4	33
8	No clear exhalation technique points	3	4	3	4	3	5	5	3	5	4	39
9	Lack of pre‐examination education process	4	5	3	5	5	4	5	5	3	3	42
10	PFT device not calibrated	2	2	4	5	3	4	4	4	4	3	35
11	Unreasonable report design	5	5	5	5	3	3	3	3	4	3	39
12	Noisy environment	1	2	2	5	3	1	3	3	1	2	23
13	No separate operation area	2	3	3	5	3	4	3	3	5	3	34

*Note:* 10 team members scored each factor on a scale from 1 (lowest influence) to 5 (highest influence). The maximum possible score was 50 points. Based on the 80/20 principle, a threshold of ≥ 40 points (representing 80% of the maximum score of 50) was applied; factors with total scores ≥ 40 were identified as key contributors to noncompliance.

**TABLE 4:** Verification of root causes associated with noncompliant pulmonary function tests.

Item	Verification of lack of training	Verification of absence of continuous supervision	Verification of lack of pre‐examination education process
Name	Description	Description	Description
Methods	Survey and analysis	On‐site verification	On‐site verification
Criteria	The project leader reviewed relevant policies and regulations in the tertiary hospital and the three branches of the PDCA‐implementing consortium; no PFT training or assessment system was found.	No pulmonary function testing supervision or management system was found at the tertiary hospital.	No standardized pre‐examination workflow existed for pulmonary function testing in the tertiary hospital or its branches.
Verification	Among 11 operators at the branch hospitals, only 2 reported receiving training; 9 operators (81.8%) were untrained.	Verified by Gu Yongping and Shen Linfeng; confirmed absence of pulmonary function testing supervision system.	Verified on‐site by Chen Liyan and Hu Wenyan; confirmed absence of a standardized pre‐examination workflow.
Confirmed as root cause	Yes	Yes	Yes

### 2.5 Development of Countermeasures Based on the PDCA Framework

#### 2.5.1 Improvement of the Training System Using a 3 + X Individualized Training Model

##### 2.5.1.1 Small Group Classroom Training

In‐person, small group training sessions were organized in designated meeting areas to provide instruction on theoretical knowledge and technical skills. Training content included respiratory physiology, pathophysiology of respiratory diseases, PFT methodologies, and interpretation of spirometric data.

##### 2.5.1.2 On Site Practical Skills Training and Assessment

Practical hands‐on training was conducted using PFT equipment to ensure operational proficiency. Following completion of practical training, on‐site competency assessments were performed.

##### 2.5.1.3 Continuing Education

To facilitate ongoing knowledge advancement and skill maintenance, participants were required to complete a specified volume of continuing education activities. Regular theoretical and practical assessments were conducted to evaluate competency.

##### 2.5.1.4 Online Theoretical Training and Guidance

Online learning platforms were used to deliver theoretical training on PFT procedures, screening and management strategies for common chronic respiratory diseases, and long‐term monitoring of chronic respiratory conditions. Online question and answer sessions were provided to address trainee inquiries.

#### 2.5.2 Establishment of a Quality Control Team and Improvement of Quality Management Mechanisms

A PFT quality control management team was formally established within the PDCA consortium (Figure S1). Specific responsibilities were assigned to each member. Quality control personnel conducted routine, dynamic quality inspections of PFT reports through the Qiyi platform. Identified issues were promptly communicated, feedback was provided, and recommendations for corrective actions were proposed. In addition, quarterly consortium‐level meetings were convened to review feedback, implement corrective actions, and monitor trends in PFT quality indicators.

#### 2.5.3 Implementation of Pre‐Examination Procedures

To accommodate the operational characteristics of primary healthcare settings, two versions of instructional videos on PFT were developed, one in Mandarin and one in the local dialect. These videos were displayed on educational televisions in clinic corridors and on laptops in PFT rooms. QR codes were provided to allow participants to access the videos independently.

**FIGURE 4:**
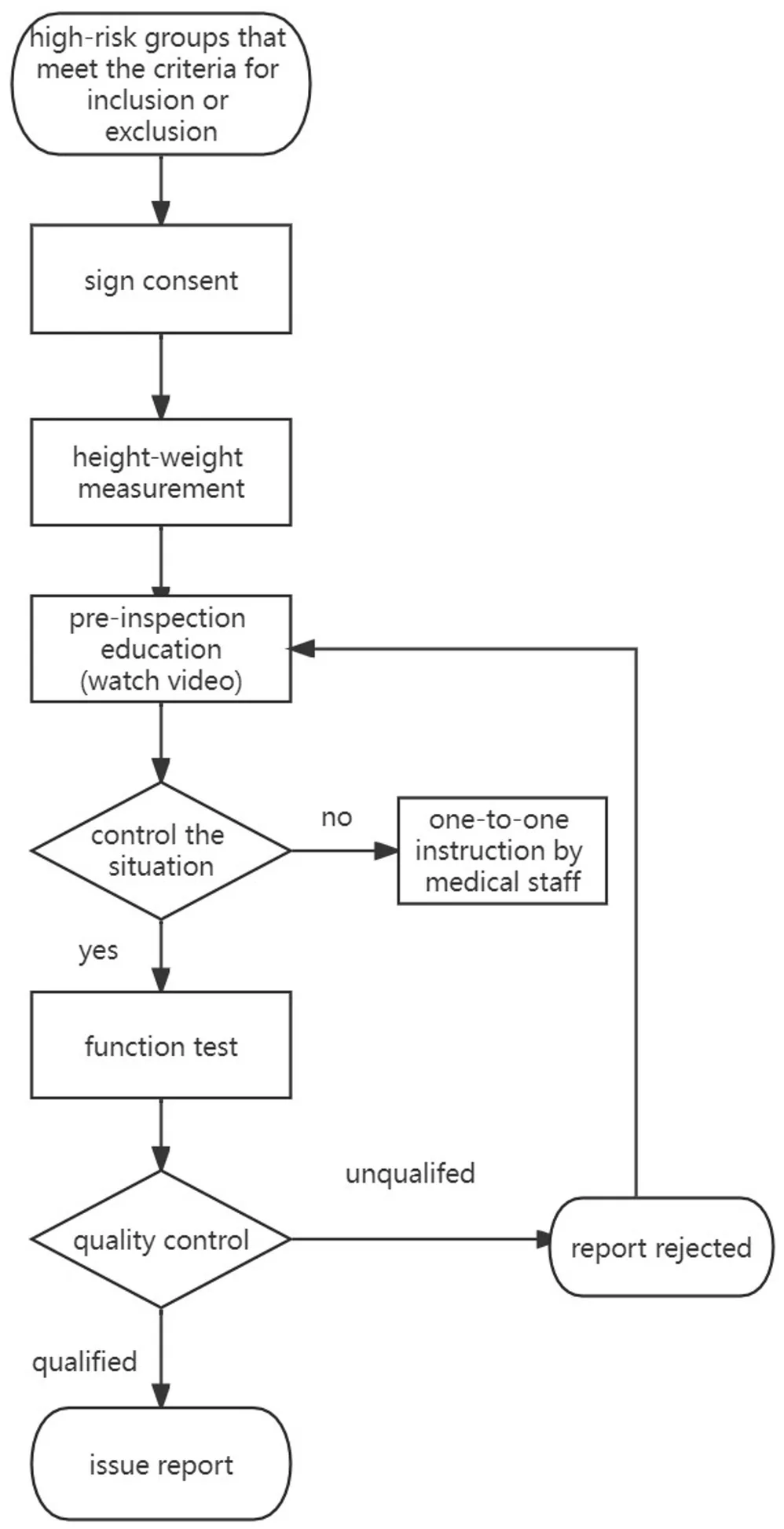
Pulmonary function testing workflow after PDCA cycle implementation.

The pre‐examination workflow was formulated in accordance with the Notice on the Technical Program for Chronic Obstructive Pulmonary Disease Screening in Zhejiang Province issued by the Zhejiang Provincial Health Commission (Zhe Wei Ban Ji Kong [2022] No. 2) and adapted to the specific conditions of primary healthcare institutions (Figure 4).

For comparison, a separate non‐PDCA consortium in the same region served as a reference group. The consortium was selected due to its analogous regional context, similar institutional level (community health service center), and equivalent spirometry equipment model. Prior to the study period, operators in the non‐PDCA consortium received basic training in pulmonary function testing (PFT) knowledge; however, they were not subjected to any structured competency assessment, ongoing supervision, or standardized pre‐examination workflow. During the observation period, no formal quality control team or PDCA‐based management system was implemented in this consortium. These baseline conditions were verified through on‐site assessments conducted by two study team members before the intervention phase. Although formal matching of individual patient characteristics was not conducted—aligning with the nature of quality improvement (QI) initiatives where the focus is on the testing process rather than individual patients—the two consortia catered to comparable community‐based COPD screening populations under the same provincial screening program (Zhe Wei Ban Ji Kong [2022] No. 2).

### 2.6 Statistical Methods

Statistical analyses were performed using SPSS version 26.0. Categorical variables, including the pass rate of pulmonary function tests and other assessment indicators, were expressed as frequencies (*n*) and percentages (%). Between‐group comparisons were conducted using the chi‐square (χ^2^) test, with Fisher's exact test applied when the expected frequency was less than 5. Pre‐ versus post‐intervention comparisons within the same group were also analyzed using the chi‐square test. All statistical tests were two‐sided, and a *p*‐value of < 0.05 was considered statistically significant.

## 3 Results

Between April 25 and June 25, 2022, 81 PFTs were conducted in the PDCA consortium, with 49.38% achieving grade C or higher. In the non‐PDCA consortium, 70 PFTs were conducted, with a 44.29% qualification rate. Insufficient expiratory effort was the main issue, causing 82.50% of noncompliance in the PDCA group and 84.50% in the non‐PDCA group. The root causes were inadequate training, lack of supervision, and no standardized pre‐exam procedures. Following implementation of PDCA cycle management approach, three standardized protocols were established and formalized as official documents: (1) the PFT training system, (2) defined roles and responsibilities of the PFT quality control management team, and (3) the standardized pre‐examination workflow for PFT. Following the implementation of these three countermeasures, the qualification rate in the PDCA consortium increased from a pre‐intervention mean of 49.38%–95.08% in October, and further increased to 96.97% in November. The qualification rate subsequently remained consistently above 95%, representing a statistically significant improvement compared to the baseline (χ^2^ = 46.72, *p* < 0.001). In contrast, the qualification rates in the non‐PDCA consortium were 55.78%, 46.98%, and 52.90% in September, October, and November, respectively. A statistically significant difference was observed between the two consortia (χ^2^ = 39.14, *p* < 0.001), which further indicates the effectiveness of the PDCA cycle management approach.

Implementation of the quality improvement initiative through multiple coordinated strategies promoted standardization of PFT quality management practices within the PDCA consortium and enhanced testing capacity in primary healthcare institutions. Individuals residing in the Qiaosi, Yunhe, and Xingqiao communities were able to obtain access to high‐quality PFT services locally, contributing to a favorable community response. The total number of pulmonary function tests increased by 105.81% compared with the previous year.

## 4 Discussion

PFT is regarded as the gold standard for the diagnosis of COPD, and test quality directly influences the effectiveness of early disease screening and the accuracy of subsequent clinical decision making processes [9]. In the present study, the implementation of a systematic PDCA cycle management approach within a regional medical consortium resulted in a significant improvement in the PFT qualification rate from 49.38% to above 95%. This outcome was significantly higher than that observed in the comparison hospital that did not implement PDCA management strategies (*p* < 0.001). These findings are consistent with those reported by Liao et al., who demonstrated improvement in PFT quality following PDCA‐based interventions, reinforcing the utility of the PDCA framework in promoting standardized healthcare quality management [10]. From a mechanistic perspective, the interventions implemented in this study addressed three root causes that were identified and verified through root cause analysis: lack of training, absence of continuous supervision, and absence of standardized pre‐examination procedures. These factors are consistent with the conclusions of Zheng et al., who indicated that defects in PFT quality mainly arise from non‐standardized operational processes and inadequate professional competence among healthcare personnel [11]. To address these gaps, the 3 + X individualized training model addressed technical skill gaps; a dedicated quality control team ensured continuous and dynamic supervision; and multilingual instructional videos optimized the pre‐examination process. Collectively, these measures formed a closed loop management framework encompassing training, execution, supervision, and improvement, which effectively mitigated common issues such as inconsistent operational practices and insufficient quality control in primary healthcare settings [12].

From a clinical perspective, improvement in test quality not only increased qualification rates but also facilitated effective implementation of early COPD screening protocols. The number of pulmonary function tests performed increased by 105.81% compared with the previous year, enabling a greater number of high‐risk individuals to undergo standardized testing and achieve early diagnosis, reflecting improved public awareness of PFT. This expansion effectively supported early detection, diagnosis, and timely intervention for individuals at risk of or living with COPD. This outcome closely aligns with the objective of improving early diagnosis of COPD as outlined in the Healthy China Action (2019–2030) initiative [2].

International comparisons further highlight the gap in PFT coverage. For instance, a cross‐sectional study conducted in Germany reported that 81.9% of patients with COPD underwent regular PFT, enabling dynamic disease monitoring and informed optimization of treatment strategies [12]. In contrast, data from China indicate that among populations aged 40 years and older, PFT coverage remains below 20%, and qualification rates at primary healthcare institutions are generally below 50% [13]. Through integration of tertiary‐level technical resources with community health service infrastructure, the present study leveraged the medical consortium model to deliver high‐quality PFT services directly to community residents. This model offers a practical reference for addressing the shortage of respiratory disease prevention and treatment resources at the primary care level in China. These findings are consistent with the conclusion of Wu et al., who emphasized the importance of coordinated quality control within medical consortia as a key strategy for improving PFT quality in primary healthcare settings [12].

### 4.1 Limitations of the Study Methods

Despite the favorable outcomes, several methodological limitations should be acknowledged. First, the sample size was relatively small, and regional representativeness was limited. The study included one core hospital in Linping District and three community health service centers. A total of 81 pulmonary function tests were conducted in the pre‐intervention phase, and the comparison hospital consisted of 70 tests. These figures fall substantially below the sample size required for multicenter studies [14]. A limited sample size may introduce bias, and the observed improvements may partially reflect random variation rather than true quality trends.

Second, certain evaluation indicators involved subjective assessment. Although an objective criterion of extrapolated volume > 0.15 L was applied to identify insufficient expiratory effort, assessment of the effectiveness of pre‐examination education, such as participants' mastery of the proper exhalation techniques, relied on judgment by healthcare personnel. The lack of standardized, objective assessment tools, such as an expiratory maneuver scoring scale, may have introduced information bias.

Third, long‐term follow‐up data were not available. Outcomes were evaluated only for 3 months following implementation, from September to November. As such, the sustainability of qualification rates remains uncertain. Therefore, the potential for short‐term improvement with subsequent regression cannot be excluded. Fourth, the non‐PDCA consortium was not systematically matched to the PDCA consortium concerning individual patient characteristics or operator‐level variables. Variations in patient age distribution, comorbidity burden, operator experience, and equipment calibration status may have influenced the observed differences in qualification rates. These factors represent potential confounders that cannot be entirely eliminated within the framework of a quality improvement initiative lacking a randomized design. Future research should consider employing propensity score matching or multicenter controlled designs to enhance causal inference. This limitation differs from the perspective emphasized by Tan et al., who indicated that PFT quality control requires sustained long‐term dynamic monitoring [15]. Future studies should incorporate extended follow‐up to verify durability of the observed effects.

### 4.2 Impact of Regional, Cultural, and Resource Factors on PDCA Cycle Implementation

The effectiveness of PDCA cycle‐based quality improvement is influenced by regional medical resource allocation, cultural context, and levels of health awareness. From a regional perspective, Linping District, located within Hangzhou, benefits from relatively strong linkage between tertiary hospitals and community health service centers, and access to real time quality control systems such as the Qiyi platform. These factors facilitate effective implementation of the Check phase of the PDCA cycle. In contrast, in less developed central and western regions, primary healthcare institutions may lack digital infrastructure and receive limited technical support from tertiary hospitals [16]. For example, a survey of community health service centers in a county of Guizhou Province reported that only 32.5% of community health service centers were equipped with network enabled PFT devices, preventing real‐time report review [17]. This contrasts markedly with the daily dynamic quality control conditions achieved in the present study. From a cultural perspective, educational interventions in this study were adapted to the local context by producing instructional videos in both Mandarin and the local dialect, which effectively addressed problems related to misunderstanding of testing instructions.

### 4.3 PDCA Long‐Term Maintenance Mechanism and Cost‐Effectiveness Analysis

The sustainability of PDCA cycle implementation depends on the establishment of long‐term maintenance mechanisms, efficient allocation of human and material resources, and balance between cost and effectiveness. In the present study, standardized documents such as the PFT training system and defined responsibilities of the quality control team were established. However, specific strategies for regular system updates and management of staff turnover were not defined. Primary healthcare institutions often experience annual staff turnover rates of approximately 15%–20% [18]. The absence of timely training for newly recruited personnel may compromise PFT quality over time. Establishment of routine training mechanisms, including mandatory pre‐employment training and annual refresher courses, as well as integration of PFT quality indicators into performance evaluations is necessary. Evidence from a medical consortium in Shenzhen demonstrated that linking PFT qualification rates to performance‐based incentives increased testing quality levels to 89.7%, providing a useful reference for long‐term quality maintenance [19].

## 5 Conclusion

In summary, the application of the PDCA cycle demonstrated substantial value in enhancing the quality control of PFT for early screening of COPD. Additional research is necessary to assess the sustainability and generalizability of this quality improvement methodology.

## Author Contributions

**Tingyan Li:** conception and design of the research. **Tingyan Li, Wenyan Hu:** acquisition of data. **Lu Xu:** analysis and interpretation of the data. **Wenyan Hu, Lu Xu:** statistical analysis. **Yongping Gu:** obtaining financing; writing of the manuscript. **Tingyan Li:** critical revision of the manuscript for intellectual content. All authors read and approved the final draft.

## Funding

This work was supported by Zhejiang Province Medical and Health Science and Technology Planning Project (NO. 2023XY008).

## Ethics Statement

The authors have nothing to report.

## Consent

The authors have nothing to report.

## Conflicts of Interest

The authors declare no conflicts of interest.

## Supporting information


**Figure S1:** Organizational framework of the pulmonary function testing quality control team.

## Data Availability

The datasets used and/or analyzed during the current study are available from the corresponding author upon reasonable request.
